# Anti-influenza Virus Activity of Methylthio-Formycin Distinct From That of T-705

**DOI:** 10.3389/fmicb.2022.802671

**Published:** 2022-02-18

**Authors:** Naoki Takizawa, Hisashi Takada, Maya Umekita, Masayuki Igarashi, Yoshiaki Takahashi

**Affiliations:** Institute of Microbial Chemistry (BIKAKEN), Tokyo, Japan

**Keywords:** antiviral compound, anti-influenza virus compound, nucleoside analog, natural product, formycin

## Abstract

Seasonal influenza virus epidemics result in severe illness, and occasionally influenza pandemics cause significant morbidity and mortality, although vaccines and anti-influenza virus drugs are available. By screening an in-house library, we identified methylthio-formycin (SMeFM), an adenosine analog, as a potent inhibitor of influenza virus propagation. SMeFM inhibited the propagation of influenza A and B viruses (IC_50_: 34.1 and 37.9 nM, respectively) and viruses showing reduced susceptibility to baloxavir and neuraminidase inhibitors but not T-705 (Favipiravir). However, the combination of T-705 and SMeFM inhibited the propagation of the influenza virus not in an antagonistic but in a slightly synergistic manner, suggesting that SMeFM has targets distinct from that of T-705. SMeFM induced A-to-C transversion mutations in virus genome RNA, and SMeFM triphosphate did not inhibit *in vitro* viral RNA synthesis. Our results show that SMeFM inhibits the propagation of the influenza virus by a mechanism different from that of T-705 and is a potential drug candidate to develop for anti-influenza drug.

## Introduction

Influenza virus infection is a threat to global health due to significant morbidity and mortality. Seasonal influenza is estimated to affect about 5–20% of the population, resulting in approximately 3 to 5 million severe illnesses globally (Peasah et al., 2013; World Health Organization, 2014). In addition to seasonal epidemics, influenza pandemics occur every 20–30 years caused by the new or re-emerging influenza A virus. Despite the existence of vaccines and antiviral drugs against influenza viruses, influenza epidemics and pandemics still emerge. To stop annual epidemics and future pandemics, effective vaccines and antiviral drugs against influenza virus infections are still required.

Influenza A and B viruses (IAV and IBV, respectively) possess eight single-stranded negative-sense RNAs as their genome (viral RNA: vRNA) (Noda et al., 2006; Nakatsu et al., 2016). In infected cells and virions, vRNA forms viral ribonucleoprotein complexes (vRNP) with heterotrimeric viral RNA polymerase proteins (PB1, PB2, and PA) and nucleoprotein (NP) (Weis and te Velthuis, 2021). The transcription and replication of the viral genome occur in the nucleus of infected cells. Viral RNA polymerases cannot synthesize cap structure *de novo* and thus perform cap-snatching from newly synthesized host pre-mRNAs. The capped RNA oligonucleotides are used as primers for viral mRNA synthesis. Viral RNA polymerases replicate vRNA *via* a complementary RNA (cRNA) intermediate by the primer-independent reaction. cRNAs are synthesized from vRNA, and then progeny vRNAs are synthesized from cRNA as a template.

Several anti-influenza drugs have been approved and are undergoing clinical trials. Amantadine and rimantadine are matrix protein 2 (M2) inhibitors (Davies et al., 1964; Wingfield et al., 1969) but M2 inhibitors are not recommended to use due to resistance in current seasonal and some of the avian subtype viruses. Zanamivir, oseltamivir, laninamivir, and peramivir are neuraminidase (NA) inhibitors (von Itzstein et al., 1993; Eisenberg et al., 1997; Kim et al., 1997; Smee et al., 2001; Yamashita et al., 2009). Baloxavir marboxil is a PA inhibitor targeting cap-dependent endonuclease activity (Hayden et al., 2018; Noshi et al., 2018; Omoto et al., 2018). Nucleotide analogs have been developed as antiviral drugs against a variety of viruses. Ribavirin and T-705 (Favipiravir) are well-known nucleotide analog and nucleobase analog with anti-influenza activity (Sidwell et al., 1972; Furuta et al., 2002a). T-705 was approved in Japan in 2014 for restricted use only when an influenza pandemic occurs by novel or re-emerging influenza virus because of its association to cause teratogenicity and embryotoxicity. The ribosyl-triphosphate form of T-705 (T-705-RTP) and ribavirin triphosphate potently or slightly inhibit influenza virus polymerase activity, respectively (Eriksson et al., 1977; Wray et al., 1985a; Furuta et al., 2005; Jin et al., 2013). Moreover, the cell culture passaging of influenza virus with T-705 or ribavirin leads to virus mutagenesis (Wray et al., 1985b; Baranovich et al., 2013; Jin et al., 2013; Sangawa et al., 2013; Vanderlinden et al., 2016). Ribavirin monophosphate inhibits the cellular IMP dehydrogenase (IMPDH), which is part of the *de novo* purine nucleotide biosynthesis pathway (Wray et al., 1985b). This inhibition activity reduces the cellular GTP pool and may induce the virus mutagenic effect. On the other hand, T-705-RTP is recognized by viral polymerases as a pseudo substrate of GTP and ATP and incorporated into RNAs synthesized by viral polymerases (Jin et al., 2013; Sangawa et al., 2013). Due to its rotating carboxamide, T-705 mimics guanine and adenine, and hence, T-705 induces mutations in the viral genome. Under 20 μM T-705, mutations in viral RNA are considered to be the main effect of inhibition of viral propagation rather than direct inhibition of viral polymerase (Vanderlinden et al., 2016).

A clinical trial of ribavirin indicated that ribavirin is not very effective against the influenza virus (Magnussen et al., 1977), and T-705 has been approved for use against only pandemic influenza virus in Japan. Nonetheless, ribavirin and T-705 are considered for use in other viral infections since these drugs have broad-spectrum antiviral activity. Remdesivir which has an *in vitro* antiviral effect against SARS-CoV-2 and is approved for treatment against COVID-19 was originally developed to treat hepatitis C. Later, remdesivir was developed for an anti-Ebola virus drug, and clinical trials were conducted (Warren et al., 2016; Mulangu et al., 2019). These cases show that nucleotide analogs have the potential to act as broad-spectrum antiviral compounds to prepare for the next pandemic. Here, we found that methylthio-formycin (SMeFM), a nucleoside analog semisynthesized from formycin, possesses strong anti-influenza virus activity. It has been reported that formycin A has an antiviral activity for IAV (WSN strain), poliovirus, vaccinia virus, and VSV (Takeuchi et al., 1966; Ishida et al., 1967) and that SMeFM has anti-influenza virus activity (Takeuchi et al., 1967; Kunimoto et al., 1968). However, the anti-influenza virus activity of SMeFM was shown only with qualitative results from one strain and cytotoxicity of SMeFM was not evaluated. Moreover, the mechanism of antiviral activity of SMeFM was not analyzed yet. To evaluate the anti-influenza activity of SMeFM, we quantitatively measured the propagation of IAVs and IBVs including viruses showing reduced susceptibility to approved anti-influenza drugs in the presence of SMeFM and compared the anti-influenza activity of SMeFM with that of T-705.

## Results

### Identification of SMeFM as an Anti-influenza Virus Compound

**FIGURE 1:**
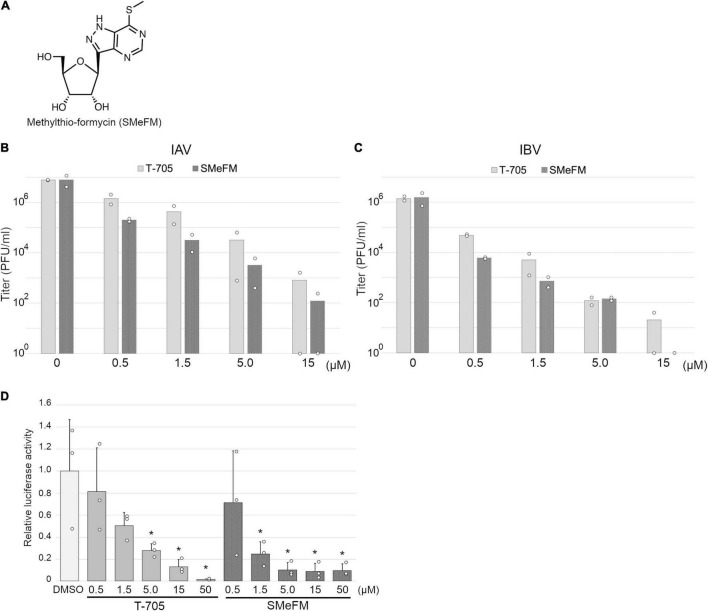
Inhibition of influenza viral polymerase activity by methylthio-formycin (SMeFM). **(A)** Chemical structure of SMeFM. **(B,C)** Inhibition of influenza virus propagation by SMeFM. MDCK cells were infected with influenza A [WSN strain; **(B)**] or B [Lee strain; **(C)**] virus at an MOI of 0.01 PFU/ml and incubated with SMeFM or T-705 for 48 h. The virus titer in the supernatant was determined by plaque assay. The bar indicates the average titer of two independent experiments. The circles indicate the titer of each experiment. The circles of 10^0^ are below the detection limit (<40 PFU/ml). **(D)** Viral polymerase activity in the presence of T-705 or SMeFM. Mini-replicon assay was performed by viral polymerase and NP derived from PR8. After 6 h transfection, SMeFM or T-705 was added to the cell culture medium and incubated for 18 h further. The graph indicates average values with standard deviations from three independent experiments. The circles indicate the relative luciferase activity of each experiment. *P*-values were calculated by Dunnett’s multiple comparison test. An asterisk indicates *P*-value less than 0.05.

For screening new compounds that have an anti-influenza virus activity, we took advantage of an in-house natural product library (approximately 2,500 compounds that contains natural products and its derivatives). MDCK cells were infected with the IAV (WSN strain), and the compound was added to the medium (final 6 μM). By the screening, we identified SMeFM as a candidate for the anti-influenza virus compound (Figure 1A). First, we quantified cytotoxicity and the antiviral activity of SMeFM for IAV and IBV. The anti-IAV activity of SMeFM (IC_50_: 34.1 nM) was comparable to that of formycin A (IC_50_: 37.3 nM) (Table 1). The cytotoxicity of SMeFM for MDCK cells was lower than that of formycin A (Table 1). The cytotoxicity of SMeFM for human lung adenocarcinoma A549 and Calu-3 cells was comparable with or lower than that for MDCK cells, while anti-influenza virus activity of SMeFM in those cells was comparable with that in MDCK cells (Table 1). SMeFM inhibited the propagation of A/equine/2/Miami/63 strain with a distant lineage from human IAV (IC_50_: 43.8 nM) and B/Lee/40 strain (IC_50_: 37.9 nM) (Table 1). Moreover, SMeFM also inhibited the propagation of recent human IAV (H1N1pdm09 and H3N2) and IBV (Victoria and Yamagata lineages) viruses with IC_50_ in the 24–60 nM range (Table 2). These results suggest that SMeFM inhibits the propagation of wide range of strains of IAV and IBV. Next, we compared the anti-influenza virus activity of SMeFM with that of T-705. T-705 is a pyrazine analog and inhibits the propagation of broad-spectrum RNA viruses, including IAV and IBV, through a combination of chain termination and lethal mutagenesis. IC_50_ of T-705 was determined to be 200 nM (Table 1). To compare the anti-influenza virus activity of SMeFM and T-705, IAV and IBV propagation in the presence of each compound were analyzed. IC_90_ of SMeFM and T-705 for IAV and IBV was estimated from the virus titer of each concentration (Figures 1B,C). IC_90_ of SMeFM for IAV (212 nM) and IBV (90.9 nM) was lower than that of T-705 (IAV: 887 nM, IBV: 290 nM). These results suggest that the anti-IAV and IBV activities of SMeFM were higher than that of T-705.

**TABLE 1:** IC_50_ and CC_50_ of formycin A, SMeFM, and T-705 for the propagation of influenza virus strains and for cell viability.

Compound	Strain	Cell	IC_50_ (nM)*	CC_50_ (μM)^#^
Formycin A	A/WSN/33	MDCK	37.3 ± 21.5	
		MDCK		2.77 ± 0.17
SMeFM	A/WSN/33	MDCK	34.1 ± 27.4	
	A/equine/2/Miami/63	MDCK	43.8 ± 27.7	
	B/Lee/40	MDCK	37.9 ± 26.6	
		MDCK		14.6 ± 9.7
	A/WSN/33	A549	52.4 ± 1.1	
		A549		20.8 ± 7.7
	A/WSN/33	Calu-3	24.4 ± 6.3	
		Calu-3		145.8 ± 41.5
T-705	A/WSN/33	MDCK	200.3 ± 11.7	
		MDCK		>1,000

*The half-maximal inhibitory concentration (IC_50_) was determined from the result of the plaque assay. Average values with standard deviations from three independent experiments were shown.

*^#^The half-maximal cytotoxic concentration (CC_50_) was determined from the result of the MTS assay.*

*Average values with standard deviations from three independent experiments were shown.*

**TABLE 2:** IC_50_ of SMeFM for the propagation of influenza virus clinical isolates.

Strain	IC_50_ (nM)*
A/KANAGAWA/ZC1802/2018 (H1N1pdm09)	32.1 ± 5.0
A/KANAGAWA/AC1877/2019 (H3N2)	23.6 ± 11.2
B/KANAGAWA/AC1807/2019 (Victoria)	60.0 ± 49.2
B/KANAGAWA/IC1740/2018 (Yamagata)	47.8 ± 19.9

*The half-maximal inhibitory concentration (IC_50_) was determined from the result of the plaque assay. Average values with standard deviations from three independent experiments were shown.

To investigate the inhibition of viral polymerase activity by SMeFM, a mini-replicon assay was performed. The viral polymerases and NP expression vectors, and vRNA encoding firefly luciferase expression vector were transfected into HEK293T cells, and SMeFM or T-705 was added to the medium after 6 h transfection. The relative luciferase activity was decreased by adding T-705 and SMeFM in a dose-dependent manner (Figure 1D). These results suggest that SMeFM inhibits influenza viral polymerase activity.

### Antiviral Activity of SMeFM for Viruses Resistant to Existing Anti-influenza Virus Drugs

**FIGURE 2:**
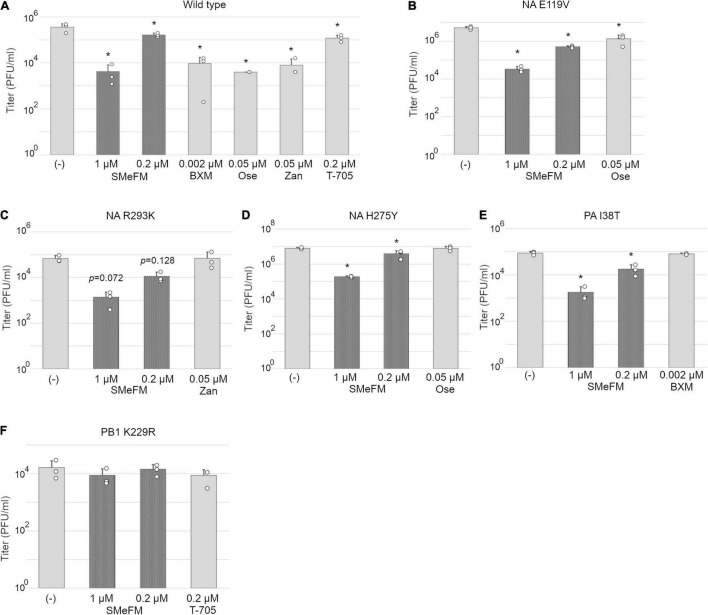
PB1 K229R that reduces susceptibility to T-705 also reduces susceptibility to SMeFM. MDCK cells were infected with wild type (PR8 strain) **(A)** or mutant virus containing NA E119V **(B)**, NA R293K **(C)**, and H275Y **(D)** (N1 numbering; low susceptibility to neuraminidase inhibitors), PA I38T **(E)** (low susceptibility to baloxavir marboxil), or PB1 K229R **(F)** (low susceptibility to T-705) at an MOI of 0.01 and incubated with SMeFM or indicated compound for 24 h. The virus titer in the supernatant was determined by plaque assay. BXM: baloxavir marboxil, Ose: oseltamivir, and Zan: zanamivir. The graph indicates average values with standard deviations from three independent experiments. The circles indicate the titer of each experiment. *P*-values were calculated by Dunnett’s multiple comparison test. An asterisk indicates *P*-value less than 0.05.

To characterize the antiviral activity of SMeFM and to evaluate the potential of a new anti-influenza virus drug, we analyzed whether SMeFM inhibits the propagation of viruses resistant to existing anti-influenza virus drugs. For strains resistant to NA inhibitors, three amino acid substitutions (E119V, H275Y, and R293K; N1 numbering) were selected based on the summary of NA amino acid substitutions associated with reduced inhibition by neuraminidase inhibitors published by WHO (World Health Organization, 2020). NA E119V and H275Y confer reduced inhibition (RI)/highly reduced inhibition (HRI) and HRI to oseltamivir and NA R293K confers RI to zanamivir in H1N1 background. Virus strains with PA I38T amino acid substitutions were clinically isolated as low-sensitivity strains to baloxavir marboxil (Hayden et al., 2018; Omoto et al., 2018). We constructed recombinant viruses containing each amino acid substitution, and the propagation of these recombinant viruses in the presence of SMeFM was analyzed. Oseltamivir, zanamivir, and SMeFM inhibited the propagation of the wild type virus at 0.05, 0.05, and 0.2 μM, respectively (Figure 2A). SMeFM inhibited the propagation of the recombinant virus containing NA E119V, NA R293K, NA H275Y, and PA I38T (Figures 2B–E). To evaluate the anti-influenza virus activity of SMeFM for viruses showing reduced susceptibility to approved anti-influenza drugs, we quantified the antiviral activity of SMeFM for recent IAV strains that has an amino acid substitution of NA E119V (H3N2), NA H275Y (H1N1pdm09), and PA I38T (H1N1pdm09). SMeFM also inhibited the propagation of these recent human IAV strains with IC_50_ in the 31–79 nM range (Table 3). These results suggest that SMeFM has an inhibitory effect on the propagation of viruses showing reduced susceptibility to NA inhibitors and baloxavir marboxil.

**TABLE 3:** IC_50_ of SMeFM for the propagation of influenza virus clinical isolates with low susceptibility to anti-influenza drugs.

Strain	IC_50_ (nM)*
A/KANAGAWA/IC1890/2019 (H1N1pdm09, PA I38T)	79.2 ± 26.6
A/KANAGAWA/ZC1905/2019 (H1N1pdm09, NA H275Y)	55.4 ± 26.7
A/KITAKYUSYU/36/2015 (H3N2, NA E119V)	31.4 ± 16.5

*The half-maximal inhibitory concentration (IC_50_) was determined from the result of the plaque assay. Average values with standard deviations from three independent experiments were shown.

**FIGURE 3:**
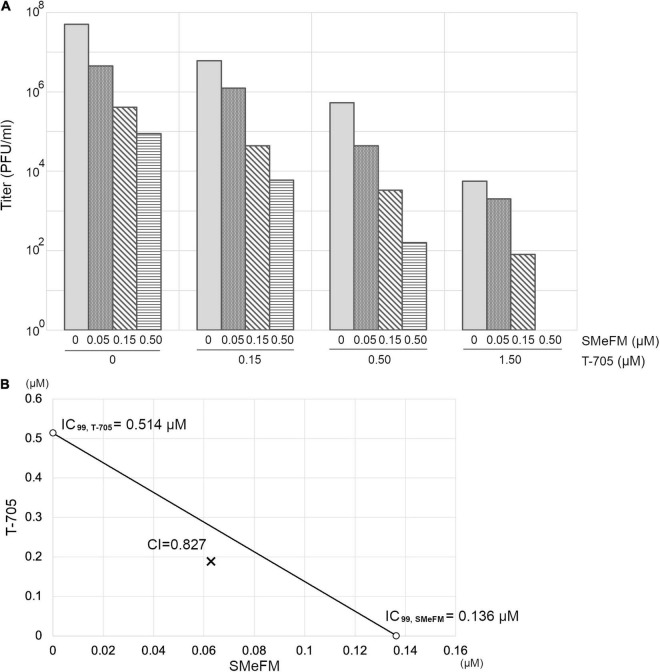
Anti-influenza virus activity of T-705 does not affect that of SMeFM. **(A)** Inhibition of IAV propagation by the combination of SMeFM and T-705. MDCK cells were infected with wild type virus (WSN strain) at an MOI of 0.01 and incubated with the indicated concentration of SMeFM and T-705 for 24 h. The virus titer in the supernatant was determined by plaque assay. **(B)** Isobologram analysis of the combination of SMeFM and T-705. The individual doses of SMeFM and T-705 to achieve 99% growth inhibition (IC_99_) were plotted on the X- and Y-axes. The line of additivity on the isobologram represents the 99% growth inhibition level of each drug. The combination index (CI) is represented by the X symbol above (CI > 1, indicating antagonism between drugs) or below (CI < 1, indicating synergy) the line (CI = 1, indicating additive effect).

We next examine the antiviral activity of SMeFM for viruses showing reduced susceptibility to T-705. Virus strain with PB1 K229R and PA P653L was isolated as a virus showing reduced susceptibility to T-705 by serial passage in cell culture, and PB1 K229R conferred resistance to T-705 (Goldhill et al., 2018). The recombinant virus containing PB1 K229R had low susceptibility to T-705, and SMeFM did not inhibit the propagation of the recombinant virus at the concentrations that inhibited the propagation of wild type virus (Figures 2A,F). These results suggest that amino acid substitution in PB1 K229R is responsible for the low susceptibility to both T-705 and SMeFM. To analyze whether the anti-influenza virus activity of SMeFM is competitive with that of T-705, both SMeFM and T-705 were added to cells infected with IAV, and the virus titer in the supernatant was determined. SMeFM inhibited the propagation of IAV in a dose-dependent manner both in the absence and presence of T-705 (Figure 3). To determine whether SMeFM and T-705 act antagonistically or synergistically, the combination index (CI) was used for evaluation. To calculate CI, we determined IC_99_s of SMeFM and T-705 from the results of Figure 3A. IC_99_s of SMeFM and T-705 were 0.136 and 0.514 μM, respectively. Next, we determined IC_99_ when SMeFM and T-705 were added simultaneously. Based on the respective IC_99_s, T-705 was considered to be as effective as SMeFM in inhibiting viral propagation at concentrations three times higher than SMeFM. Therefore, we determined the IC_99_ under the condition that the concentration ratio of SMeFM and T-705 was 1:3 from the results of Figure 3A. The IC_99_ was 0.251 μM when SMeFM and T-705 were added simultaneously. From these IC_99_s, CI was calculated by the formula described in the Materials and Methods, and the value was 0.83 (Figure 3B). These results suggest that the anti-influenza virus activities of SMeFM and T-705 are not antagonistic, but have some synergistic effects.

### Mechanism of Anti-influenza Virus Activity of SMeFM in Infected Cells

**FIGURE 4:**
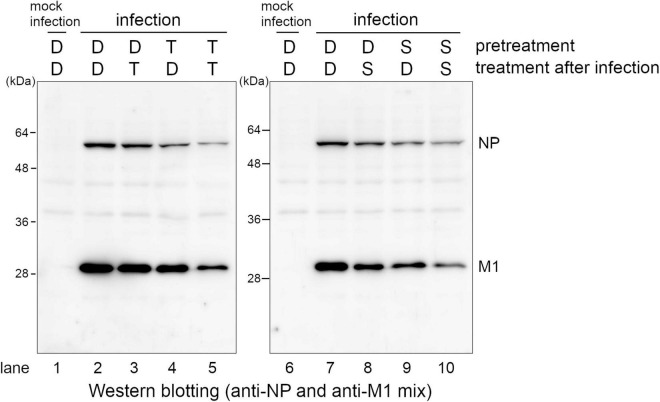
Pre-treatment of SMeFM enhances the inhibitory effect of SMeFM on viral protein synthesis. MDCK cells were pretreated with 3 μM of T-705 or 1 μM of SMeFM for 24 h. The pretreated cells were infected with the influenza A virus (WSN strain) at an MOI of 1. The cells were collected and lysed at 8 hpi, and NP and M1 were detected by western blotting. D: DMSO, T: T-705, S: SMeFM.

Next, we analyzed whether pre-treatment of SMeFM is necessary for anti-influenza virus activity of SMeFM. The cells pretreated with SMeFM or T-705 were infected, and the expression of viral proteins was detected. Since T-705 is metabolized to an active form, the expression of viral proteins was decreased in cells treated with T-705 before and after infection, and slightly decreased in cells pretreated only with T-705 (Figure 4, lanes 2, 4, and 5). The expression of viral proteins was clearly decreased in cells treated with SMeFM before and after infection (Figure 4, lanes 7 and 10), whereas that was slightly decreased in cells treated with SMeFM only after infection (Figure 4, lane 8). Moreover, the expression of viral proteins was decreased in cells pretreated only with SMeFM (Figure 4, lane 9). These results suggest that pre-treatment of SMeFM enhances anti-influenza virus activity.

**FIGURE 5:**
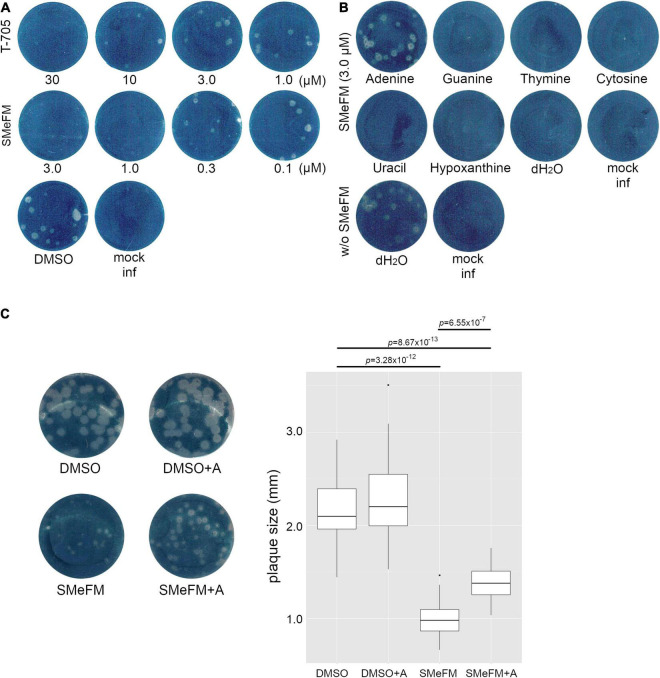
The addition of adenine attenuates the inhibitory effect of SMeFM on viral propagation. **(A)** Inhibition of plaque formation by SMeFM. MDCK cells were infected with the influenza A virus (WSN strain, 20 PFU) and were overlayed with MEM-0.8% agarose containing SMeFM or T-705. After 48 h incubation, the cells were fixed and stained by amido black. **(B)** Plaque assay with SMeFM and nucleobases. MDCK cells were infected with the influenza A virus (20 PFU) and were overlayed with MEM-0.8% agarose containing 3 μM SMeFM and an indicated nucleobase (20 μM). **(C)** The plaque size with SMeFM and adenine. The plaque of infected cells overlayed with agarose containing DMSO, both DMSO and 20 μM adenine, 1 μM SMeFM, or both SMeFM and adenine (left panel). The plaque size incubated with DMSO, both DMSO and adenine, SMeFM, or both SMeFM and adenine (*n* = 30, 30, 18, and 32, respectively) was measured and plotted (right panel). *P*-values were calculated by Wilcoxon rank sum test and were corrected by Bonferroni method.

To investigate which nucleobase analog SMeFM acts as, we performed a competition experiment. SMeFM and nucleobase were added to agarose in plaque assay, and antiviral activity of SMeFM and competition of that activity by nucleobase were determined by plaque size (Figure 5A). When adenine and SMeFM were added, the plaque size was clearly increased compared to that without adenine (Figures 5B,C). These results suggest that SMeFM acts as an adenosine analog.

**FIGURE 6:**
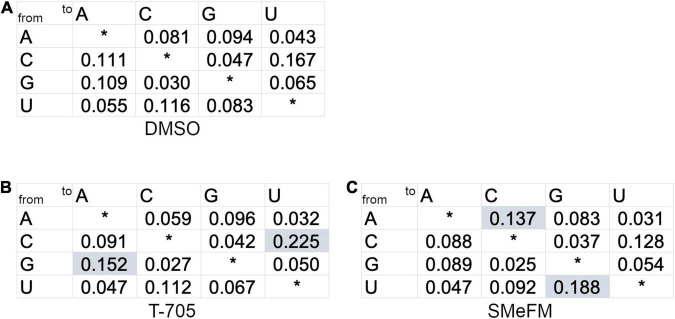
Adenine to cytosine and uracil to guanine mutations are accumulated in viral genome RNA by the treatment of SMeFM. The mutation rate from treated infected cells treated with DMSO **(A)**, T-705 **(B)**, or SMeFM **(C)** was shown. The mutation ratio was calculated from the number of polymorphisms of each nucleotide and the total number of polymorphisms (DMSO: 40,640, T-705: 57,923, and SMeFM: 33,161) detected by amplicon-seq. A chi-square test was used to determine the statistical significance of the differences of each mutation type relative to the DMSO control. Statistically significant increases in mutation rates are highlighted by shading.

Both direct inhibitions of viral RNA synthesis and induction of mutations in the viral genome are the mechanism of antiviral activity of T-705 and ribavirin. To investigate whether SMeFM can induce mutations in the influenza virus genome, the mutation rate of the viral genome in cells treated with SMeFM was determined. The viral genome was purified from infected cells treated with SMeFM at 8 h post infection (hpi), and amplicon-seq was performed. The mutation rate of each mutation type in compound-treated cells was compared to that in mock-treated cells by a chi-square test (Figure 6). The mutation rates of C-to-U and G-to-A in cells treated with T-705 were statistically increased compared with those in mock-treated cells (Figures 6A,B). This result is consistent with the finding that T-705 acts predominantly as a guanine analog. If SMeFM acts as an adenosine analog and is incorporated into viral RNA, the mutation rates of U-to-C and A-to-G (transition mutations) are thought to be increased by pairing the base of SMeFM with cytosine. In cells treated with SMeFM, the mutation rates of A-to-C and U-to-G (transversion mutations) were increased, while the mutation rates of U-to-C and A-to-G were comparable to those in mock-treated cells (Figures 6A,C). These results suggest that SMeFM does not induce mutations directly as an adenosine analog.

### Effect of SMeFM-Triphosphate on *in vitro* RNA Synthesis Assay

**FIGURE 7:**
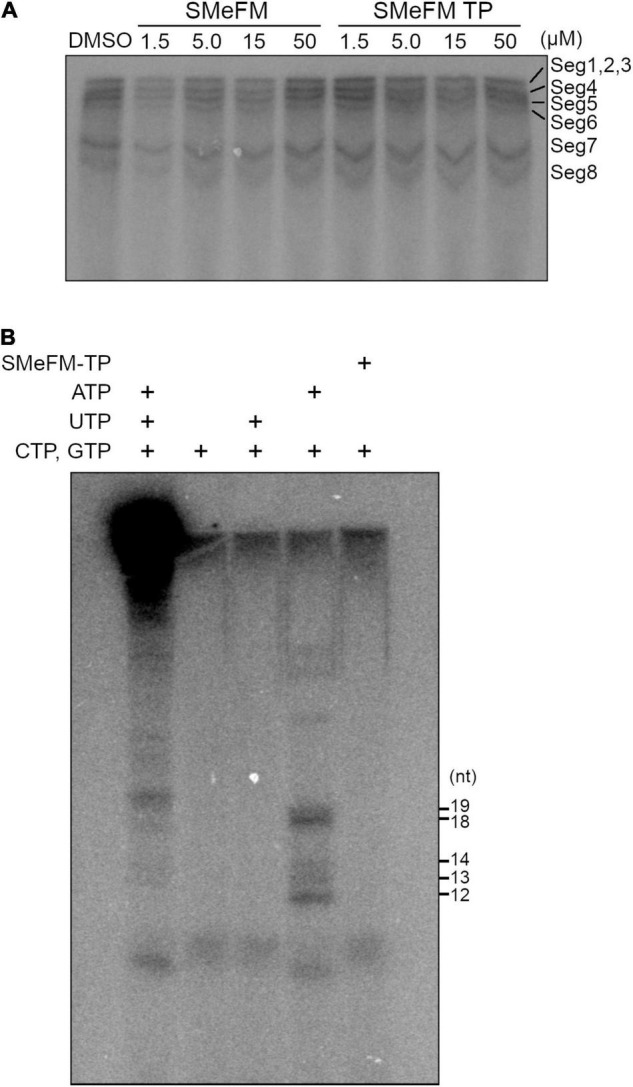
SMeFM triphosphate is not incorporated into synthesized RNA. **(A)** *In vitro* RNA synthesis from vRNPs with SMeFM and SMeFM triphosphate (SMeFM TP). *In vitro* RNA synthesis assay was performed with SMeFM or SMeFM TP at 30°C for 30 min. Synthesized RNAs were separated by UREA-PAGE. **(B)** Limited elongation assay with SMeFM TP. *In vitro* RNA synthesis assay was performed with or without UTP, ATP, or SMeFM. Synthesized RNAs were separated by UREA-PAGE. The expected lengths of limited elongation products without UTP were 12 nt for segments 1, 3, and 7, 13 nt for segment 5, 14 nt for segment 6, 18 nt for segment 4, and 19 nt for segment 2.

To analyze whether SMeFM inhibits viral RNA synthesis directly, *in vitro* transcription of viral RNA was performed. Since SMeFM is not a substrate for viral polymerase due to the lack of phosphate group, we synthesized SMeFM-triphosphate (SMeFM-TP) and investigated the inhibition of viral RNA synthesis by SMeFM and SMeFM-TP. SMeFM and SMeFM-TP did not inhibit viral RNA synthesis from purified vRNP (Figure 7A). Next, to determine whether SMeFM was incorporated into viral RNA directly, a limited elongation assay was performed. When *in vitro* transcription was performed without UTP, RNA fragment was elongated to just before the first A nucleotide in template vRNA (12 nt for segment 1, 3, and 7, 13 nt for segment 5, 14 nt for segment 6, 18 nt for segment 4, and 19 nt for segment 2) (Figure 7B). When *in vitro* transcription was performed without ATP or without UTP and ATP, elongated RNA fragment was not detected because RNA synthesis was stopped at the first U nucleotide in template vRNA (3 nt for all segment) (Figure 7B). If SMeFM-TP would be a substrate for RNA elongation instead of ATP, the elongated RNA fragment could be detected when *in vitro* transcription was performed with SMeFM-TP instead of ATP. However, when SMeFM-TP was added instead of ATP, the elongated RNA fragment was not detected (Figure 7B). These results suggest that SMeFM-TP is not incorporated into vRNA by viral polymerase as a substitute for ATP.

**FIGURE 8:**
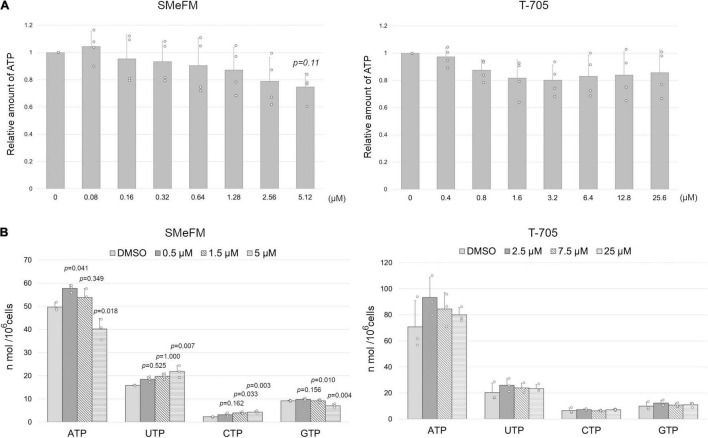
SMeFM treatment reduces the cellular ATP pool. **(A)** The relative concentration of ATP in cells treated with SMeFM. The cellular ATP concentration was measured as luminescence count and was normalized by the cell number. The relative ATP concentration was calculated by dividing the normalized luminescence count by that of cells without SMeFM treatment which is set as 1. The graph indicates average values with standard deviations from four independent experiments. *P*-values were calculated by Dunnett’s multiple comparison test. **(B)** The concentration of NTPs in cells treated with SMeFM. MDCK cells were treated with SMeFM or T-705 at indicated concentrations, and NTP concentration was analyzed by HPLC. The graph indicates average values with standard deviations from three independent experiments. The circles indicate the value of each experiment. *P*-values were calculated by Dunnett’s multiple comparison test.

It has been reported that SMeFM inhibits phosphoribosyl pyrophosphate amidotransferase, which catalyzes the first step of *de novo* purine biosynthesis and IMPDH (Crabtree et al., 1973; Shantz et al., 1973). Thus, we determined the amount of ATP in cells treated with SMeFM. The concentration of ATP was converted to luciferase activity, and the relative amount of ATP was calculated. The amount of ATP in cells treated with SMeFM appeared to decrease in a dose-dependent manner though there was no significant difference in the amount of ATP between SMeFM treated and non-treated cells when a *p*-value less than 0.05 was considered statistically significant (Figure 8A). Based on this result, we decided to directly measure nucleotide concentrations by HPLC analysis. To evaluate the relationship between viral propagation and intracellular nucleotide concentration, we measured intracellular nucleotide concentration at SMeFM and T-705 concentrations that reduce viral propagation to 1/100 to 10,000. Previous reports have shown that the addition of 20 and 60 μM T-705 reduces the amount of intracellular GTP to 80 and 60%, respectively (Vanderlinden et al., 2016). In our analysis, the addition of 25 μM T-705 did not change the intracellular GTP concentration (Figure 8B). The amounts of ATP and GTP were decreased in cells treated with 5 μM SMeFM, whereas those of UTP and CTP were increased (Figure 8B). These results suggest that SMeFM causes an NTP imbalance in the concentration range where viral propagation is sufficiently reduced.

## Discussion

We found that SMeFM has anti-influenza virus activity by the screening of our in-house chemical library and showed that SMeFM inhibits the propagation of IAVs and IBVs including viruses showing reduced susceptibility to approved anti-influenza drugs and that the mechanism of anti-influenza activity of SMeFM is different from that of a known nucleobase analog, T-705. In T-705 and ribavirin, the carboxamide moiety in the base provides them with ambiguous base-pairing properties, and hence the mutations can be introduced when T-705 or ribavirin is incorporated into the viral template RNA. In SMeFM, the methylthio moiety in the base could not make base-pairing. Moreover, A-to-C transversion induced by SMeFM treatment was not consistent with those induced by purine analogs (Figure 6). Our results also show that SMeFM-TP was not incorporated into viral RNA by *in vitro* viral RNA synthesis assay (Figure 7). Taken together, we concluded that SMeFM inhibits the cellular process involved in the IAV propagation rather than viral polymerase directly. We showed that SMeFM inhibited viral polymerase activity in a dose-dependent manner by mini-replicon assay (Figure 1D). This result can be explained by the decrease in the amount of ATP by SMeFM treatment before viral RNA synthesis starts. The concentration of ATP and GTP in cells treated with SMeFM was decreased compared with that in mock-treated cells (Figure 8). The decrease in cellular GTP levels by SMeFM treatment is supported by the report that SMeFM inhibits phosphoribosyl pyrophosphate amidotransferase and IMPDH (Crabtree et al., 1973; Shantz et al., 1973). However, anti-influenza virus activity of SMeFM was reduced by the adding of adenine in the cell culture medium and was not reduced by the adding of guanine (Figure 5). These results indicate that not GTP but ATP synthesis by salvage pathway rescue the inhibition of IAV propagation by SMeFM. In addition, pre-treatment of SMeFM enhances anti-influenza virus activity, suggesting that SMeFM is metabolized to an active form (Figure 4). SMeFM would be metabolized to SMeFM monophosphate which would inhibit the *de novo* ATP synthesis pathway from inosine monophosphate to ATP, and this inhibition is thought to suppress the IAV propagation. A 100-fold SMeFM concentration was required to reduce the cellular ATP concentration compared to the inhibition of the IAV propagation. It is speculated that influenza virus infection causes changes in ATP synthesis and ATP localization, which are inhibited by the low-concentration SMeFM treatment. The relationship between local ATP level and viral propagation needs to be elucidated in the future.

We used baloxavir marboxil instead of baloxavir acid, the active metabolite of baloxavir marboxil, for evaluating the inhibition of influenza virus propagation. Baloxavir marboxil would be metabolized to baloxavir acid by arylacetamide deacetylase and carboxylesterases (CESs) *in vivo*, and MDCK contains CESs (Di, 2019). This suggests that baloxavir marboxil is metabolized to the active form in MDCK cells. Moreover, we have confirmed baloxavir marboxil inhibited the propagation of influenza virus in MDCK cells similar to baloxavir acid (Takizawa et al., submitted). Baloxavir marboxil inhibited the propagation of wild type strain in MDCK cells (Figure 2A) and could not inhibit that of viruses showing reduced susceptibility to baloxavir marboxil (Figure 2E). These results indicate that the use of baloxavir marboxil for MDCK cells induces the same inhibition of influenza virus propagation as the use of baloxavir acid.

A IAV strain showing reduced susceptibility to T-705, which contains K229R amino acid substitution in PB1, also shows reduced susceptibility to SMeFM though the antiviral mechanism of T-705 is different from that of SMeFM (Figures 2F, 3). PB1 K229 is located in motif F of RNA dependent RNA polymerase, contributing to NTP binding (Goldhill et al., 2018). Modeling of K229R amino acid substitution showed that it reduces the NTP binding space of the active site (Goldhill et al., 2018). Reduction of the NTP binding space by the K229R affects the efficiency of NTP and T-705-triphosphate incorporation and may account for the reduction in polymerase activity. We hypothesized that SMeFM treatment causes local NTP pool imbalance and thus induces mutations in vRNA. Since the efficiency of NTP incorporation to the active site is altered in viral polymerase complex containing PB1 K229R, it is likely that mutations due to the local NTP pool imbalance are not induced in cells infected with virus containing PB1 K229R. PB1 K229R reduces the virus propagation without antiviral compound treatment. Recombinant virus containing both PB1 K229R and PA P635L shows reduced susceptibility to T-705, and the propagation of the recombinant virus is comparable to that of the wild type virus (Goldhill et al., 2018). However, in our experiment, the propagation of the recombinant virus containing both amino acid substitution from PR8 strain was still reduced compared with that of wild type virus (data not shown), and thus we used PB1 K229R virus for the assay. This result may be due to the use of different virus strains. Since the PA P653L amino acid substitution is not directly associated with the low-susceptibility to T-705, it is likely that PB1 K229R and PA P653L mutant virus is also low-susceptibility to SMeFM.

Due to high anti-influenza virus activity, SMeFM is a compound to develop as anti-influenza drug. The detailed mechanism of its anti-influenza virus activity and metabolic stability for *in vivo* analysis needs to be investigated. The potential risk associated with T-705 for teratogenicity and embryotoxicity suggests challenges with dosing for clinical use though cytotoxicity of T-705 is very low in culture cells. SMeFM may also pose such risks. Therefore, it is necessary to reduce the cytotoxicity of SMeFM by synthesizing derivatives as well as to evaluate it for teratogenicity and embryotoxicity. SMeFM is a kind of C-nucleoside in which the sugar and the base are linked through the C-C bond (De Clercq, 2015). C-nucleotides are resistant against degradation by phosphorolysis and are hydrolytically stable even with a 1″-substituent. For these reasons, C-nucleotides are promising for the development of antiviral drugs. Synthesis of SMeFM derivatives and analysis of their antiviral activity for a wide range of viruses will contribute to the development of new antiviral drugs not only for influenza viruses but for future pandemic viruses.

## Materials and Methods

### Reagents

T-705 was synthesized with reference to the patent (Furuta et al., 2002b). NMR data is described in the Supplementary Material. SMeFM and SMeFM-TP were synthesized from formycin A. The detailed synthesis method and NMR data are described in the Supplementary Material. Oseltamivir phosphate and zanamivir hydrate were purchased from FUJIFILM Wako Pure Chemical (Tokyo, Japan) and TCI (Tokyo, Japan). Baloxavir marboxil was purchased as a drug from SHIONOGI (Osaka, Japan).

### Cells and Viruses

MDCK and Calu-3 cells were maintained in a minimal essential medium (MEM) (Sigma-Aldrich, ST. Louis, MO, United States) containing 10% fetal bovine serum and penicillin/streptomycin (Nacalai Tesque, Kyoto, Japan). HEK293T and A549 cells were maintained in Dulbecco’s modified Eagle’s medium (DMEM) with high glucose concentration (Sigma-Aldrich) containing 10% fetal bovine serum and penicillin/streptomycin.

Influenza virus [A/Puerto Rico/8/34 (PR8) and A/WSN/33] and recombinant viruses based on PR8 strain were generated by reverse genetics approach (Neumann et al., 1999; Ohkura et al., 2014). Four viral protein expression vectors (Neumann et al., 1999) and eight viral RNA expression vectors derived from the PR8 strain (Ohkura et al., 2014) were transfected to HEK293T cells using polyethylenimine (Polysciences, Warrington, PA, United States). After 24 h of transfection, the culture medium was changed to OPTI-MEM I (Thermo Fisher Scientific, Waltham, MA, United States) containing 0.6 μg/ml L-1-tosylamide-2-phenylethyl chloromethyl ketone treated trypsin (TPCK-trypsin; Sigma-Aldrich). After incubation for 24 h, the cell culture supernatant was collected. The virus titer was determined by a plaque assay. pPolI-PR8 mutant vectors encoding NA E119V, NA R293K, PA I38T, and PB1 K229R were constructed by an inverted PCR. A/equine/2/Miami/63 strain and influenza B virus (B/Lee/40) were provided from ATCC (Manassas, VA, United States). A/KANAGAWA/ZC1802/2018 (H1N1pdm09), A/KANAGAWA/AC1877/2019 (H3N2), B/KANAGAWA/AC1807/2019 (Victoria), B/KANAGAWA/IC1740/2018 (Yamagata), A/KANAGAWA/IC1890/2019 (H1N1pdm09, PA I38T), A/KANAGAWA/ZC1905/2019 (H1N1pdm09, NA H275Y), and A/KITAKYUSYU/36/2015 (H3N2, NA E119V) strains were kindly provided from the National Institute of Infectious Diseases, Japan.

### Screening

MDCK cells seeded in 96-well plates (1 × 10^4^ cells/well) were infected with influenza virus (A/WSN/33) at an MOI of 0.1 PFU/cell in MEM containing TPCK-trypsin and the test compound (final 6 μM). After incubation at 37°C for 48 h, the cells were fixed with ethanol and then stained with amido black solution (1% amido black 10B, 50% methanol, and 10% acetic acid). Cell viability was determined by measuring the optical density at 600 nm (OD_600_). The relative viability of the infected cells treated with each test compound was calculated by considering the value of the untreated cells to be 1. For the second screening, IC_50_ and CC_50_ of 1*^st^* hit compound were determined. To estimate IC_50_, the viral titer of the supernatant at 24 hpi with various concentrations of the test compound was determined by plaque assay. To CC_50_, the cell viability with various concentrations of the test compound for 48 h was determined by CellTiter 96 Aqueous One Solution Cell Proliferation Assay (MTS) (Promega, Fitchburg, WI, United States). The inhibition and viability curves were calibrated using the Rodbard curve fitting tool in the Image J, and IC_50_ and CC_50_ were estimated from the fitting curve.

### Virus Propagation With Compound

MDCK cells seeded in 24-well plates were infected with IAV at an MOI of 0.01 PFU/cell in MEM and suspended in MEM containing 0.6 μg/ml TPCK-trypsin and various concentrations of the test compound. After incubation at 37°C for 24 h, the virus titer in the supernatant was determined by plaque assay.

We used IC_99_ when SMeFM and T-705 were added in the ratio of 1:3 (IC_99,SMeFM+*T–705*_) to obtain CI. IC_99_ of SMeFM and T-705 (IC_99,SMeFM_ and IC_99,T–705_) and the conversion capacity were calculated from the results of SMeFM and T-705 alone. CI was calculated by the following formula.


$$C\,I=\frac{I\,C_{99,S\,M\,e\,F\,M+T-705\,\left(S\,M\,e\,F\,M\right)}}{I\,C_{99,S\,M\,e\,F\,M}}+\frac{I\,C_{99,S\,M\,e\,F\,M+T-705\,\left(T-705\right)}}{I\,C_{99,T-705}}$$

Synergism effect, additive effect, and antagonism effect are defined by CI < 0, CI = 0, and CI > 0, respectively.

### Mini-Replicon Assay

HEK293T cells were transfected with pcDNA-PB2, pcDNA-PB1, pcDNA-PA, pCAGGS-NP, pHH-vNSLuc, and pRL-CMV (Promega) using polyethylenimine. After a 6 h transfection, SMeFM or T-705 was added to the cell culture medium, and 24 h after transfection, the cells were lysed with passive lysis buffer (Promega), followed by measurement of the activities of firefly luciferase (Fluc) and renilla luciferase (Rluc). The Fluc activity was normalized to that of the Rluc transfection control, and the relative influenza virus polymerase activity was calculated relative to that of the normalized Fluc activity of the untreated cells to be 1.

### Western Blotting

MDCK cells were pretreated with 3 μM T-705, 1 μM SMeFM, or DMSO for 24 h. The pretreated cells were infected with the influenza A virus (WSN) at an MOI of 1 PFU/cell and incubated at 37°C with 3 μM T-705, 1 μM SMeFM, or DMSO. The cells were lysed at 8 hpi with cell lysis buffer [20 mM Tris–HCl (pH 7.9), 100 mM NaCl, 1 mM EDTA, and 0.1% NP-40] on ice for 10 min. The protein concentration of the lysate was measured by Bradford protein assay reagent (Bio-Rad, Hercules, CA, United States). The protein concentration was prepared identically in each cell lysate and the cell lysate was separated by SDS-PAGE. Viral proteins were detected by western blotting with ImageQuant LAS 4000 (GE Healthcare, Milwaukee, WI, United States) using rabbit polyclonal antibodies for NP (Kawaguchi et al., 2011) and M1 (Takizawa et al., 2006).

### Targeted Amplicon Sequencing and Data Analysis

MDCK cells were pre-treated with 0.25 μM SMeFM or 0.50 μM T-705 for 24 h. The pretreated cells were infected with the influenza A virus (PR8) at an MOI of 0.1 PFU/cell for 1 h and incubated at 37°C with 0.25 μM SMeFM or 0.50 μM T-705. Total RNA was extracted using ISOGEN reagent (Nippon Gene, Tokyo, Japan) at 8 hpi, and cDNA was synthesized with the Uni12 primer using ReverTra Ace (Toyobo, Osaka, Japan). The fragment of segment 7 vRNA (nucleotide position 406–640) was amplified with specific primers using KOD One (Toyobo). A second PCR was performed with adaptor and index primers using KOD One (Toyobo). Sequencing was performed using a MiSeq sequencer (Illumina San Diego, CA, United States) with MiSeq Reagent Micro Kit v2 (300 cycles) (illumine). Read sequences were filtered according to base quality using Trimmomatic (*q* = 30) (Bolger et al., 2014). The filtered sequences were aligned, and the ratio of nucleotides in each position was calculated. A chi-square test was used to determine the statistical significance of mutation rate between DMSO treated cells and compound treated cells.

### *In vitro* Transcription of Viral RNA

The vRNP was purified from virion (PR8) as previously described (Yamanaka et al., 1990) and used as the enzyme source. The viral RNA synthesis assay was performed at 30°C for 30 min in reaction buffer (50 mM Hepes-NaOH (pH 7.4), 50 mM KCl, 3 mM MgCl_2_, 1.5 mM dithiothreitol, 100 μM each of ATP, UTP, and GTP, 20 μM CTP, 5 μCi (α-^32^P)CTP, 4 U RNase inhibitor (Takara, Shiga, Japan), and vRNP] with or without SMeFM or SMeFM-TP. The synthesized RNA was purified by phenol-chloroform extraction, separated by 6% UREA-PAGE, and then visualized using Typhoon 9400 image analyzer. For limited elongation assay, viral RNA was synthesized at 30°C for 30 min in reaction buffer without ATP and/or UTP. The synthesized RNA was purified by phenol-chloroform extraction and separated by 18% UREA-PAGE.

### Analysis of Cellular ATP and NTP Pools

The relative concentration of cellular ATP was measured by CellTiter-Glo 2.0 (Promega). MDCK cells in 96 well plates were treated with SMeFM for 24 h. CellTiter reagent was added to the cell culture medium, and the plate was incubated at room temperature for 10 min. The luminescent signal was measured by GLOMAX 20/20 Luminometer (Promega). After measurement of luminescent signal, the cells in 96 well plates were stained with amido black to measure the relative cell number, and OD_600_ was measured.

The concentration of cellular NTP pools was measured by HPLC analysis. MDCK cells in 6 cm dish were treated with SMeFM for 24 h. The cells were washed with PBS(-) and collected by trypsinization. The collected cells were counted and pelleted by centrifugation at 200 *xg* for 5 min. The cell pellet was washed with PBS(-) and suspended in 500 μl of ice-cold methanol-water (2:1). After incubation on ice for 10 min, the extracts were clarified by centrifugation at 15 k *xg* for 5 min. In these cell extracts, the nucleotide pools were separated and quantified by HPLC analysis (Alliance 2996; Waters, Milford, MA, United States) using an Xbridge Amide 3.5 μm column (2.1 mm × 150 mm; Waters). A 12-min linear gradient of 85% buffer A (25 mM ammonium carbonate, 5 mM ammonia, and 80% acetonitrile) and 15% buffer B (25 mM ammonium carbonate, 5 mM ammonia, and 40% acetonitrile) to 63% buffer A and 37% buffer B was used for separation. The UV absorbance of the peaks was recorded at 254 nm.

## Data Availability Statement

The datasets presented in this study can be found in online repositories. The names of the repository/repositories and accession number(s) can be found below: https://ddbj.nig.ac.jp/resource/sra-submission/DRA012601.

## Author Contributions

NT, MI, and YT: conceptualization. NT: formal analysis and funding acquisition. NT, HT, and MU: investigation. MI: resources. NT and HT: writing—original draft preparation. MI and YT: writing—review and editing. All authors contributed to the article and approved the submitted version.

## Conflict of Interest

The authors declare that the research was conducted in the absence of any commercial or financial relationships that could be construed as a potential conflict of interest.

## Publisher’s Note

All claims expressed in this article are solely those of the authors and do not necessarily represent those of their affiliated organizations, or those of the publisher, the editors and the reviewers. Any product that may be evaluated in this article, or claim that may be made by its manufacturer, is not guaranteed or endorsed by the publisher.
