# Supplementary material for: Anti-influenza Virus Activity of Methylthio-Formycin Distinct From That of T-705
Source: Front Microbiol. 2022 Feb 18;13:802671. doi: 10.3389/fmicb.2022.802671 (PMC8894184; doi:10.3389/fmicb.2022.802671)
Supplement: Supplementary file 1 [file Data_Sheet_1.docx]

**1. NMR spectra of T-705**

^1^H-NMR and ^13^C-NMR spectra were determined on a Brucker 600 Hz or Brucker 400 Hz instrument with TMS as an internal standard. ^19^F-NMR spectra were determined with TFT in DMSO-d6(-61.22ppm) as an external standard. ^19^F-NMR (400 MHz, DMSO-d6): δ -93.24 (s, 1F). ^1^H-NMR (600 MHz, DMSO-d6): δ 13.39 (s, OH), 8.75 (s, 1H), 8.49 (d, J = 7.9 Hz 1H), 8.47 (s, 1H), ^13^C-NMR (600 MHz, DMSO-d6): δ 168.60, 159.66, 152.30 (d, J = 244.1 Hz), 135.68 (d, J= 42.2Hz), 122.25. HRMS: m/z = 158.03 [M+H]+, 180.01 [M+Na]+.

**2. Synthesis of methylthio-formycin (SMeFM) and SMeFM triphosphate**

**General**

The reaction was performed in a pear-shaped flask or a test tube with a Teflon-coated magnetic stirring bar unless otherwise noted. Air- and moisture-sensitive liquids were transferred via a gas-tight syringe and a stainless-steel needle. All work-up and purification procedures were carried out with reagent-grade solvents under an ambient atmosphere.

**Instrumentation**

Melting points were determined on a Yanagimoto MP-S3 micro melting point apparatus and were uncorrected. Infrared (IR) spectra were recorded on a JASCO FT/IR 410 Fourier transform infrared spectrophotometer. NMR was recorded on Bruker AVANCE III HD400 and AVANCE III 500 spectrometers. Chemical shifts for proton are reported in parts per million downfield from tetramethylsilane and referenced to residual protium in the NMR solvent (CDCl3: δ 7.24 ppm, DMSO-d6: δ 2.49 ppm, D_2_O: δ 4.79 ppm). For ^13^C NMR chemical shifts are reported in the scale relative to NMR solvent (CDCl_3_: δ 77.0 ppm, DMSO-*d*_6_: δ 39.5 ppm) as an internal reference. For ^31^P NMR chemical shifts are reported in the scale relative to triphenyl phosphate (D_2_O: δ –61.2 ppm) as an external reference. NMR data are reported as follows: chemical shifts, multiplicity (s: singlet, d: doublet, dd: doublet of doublets, t: triplet, q: quartet, m: multiplet, br: broad signal), coupling constant (Hz), and integration. High-resolution mass spectra (ESI TOF (+)) were measured on ThermoFisher Scientific LTQ Orbitrap XL. Optical rotation was measured using a 2 mL cell with a 1.0 dm path length on a JASCO polarimeter P-1030.

**Materials**

Unless otherwise noted, materials and solvents were purchased from commercial suppliers and were used without purification. Silica gel column chromatography was performed with Wakogel C-200 (Spherical, 75–150 mm) purchased from FUJIFILM Wako Pure Chemical Corporation.

(2*R*,3*S*,4*R*,5*S*)-2-(Hydroxymethyl)-5-(7-mercapto-1*H*-pyrazolo[4,3-*d*]pyrimidin-3-yl)tetrahydrofuran-3,4-diol (**2**) ^ref-SMe1^

To a stirred suspension of FMA (**1**, 267 mg, 1.00 mmol) in toluene (50 mL) were added 1,2-bis[(dimethylamino)methylene]hydrazine (853 mg, 6.00 mmol) and TMSCl (758 µL, 6.00 mmol) and the mixture was stirred at 60 °C for 17 h. After removing the volatiles under reduced pressure, the resulting residue was diluted with CHCl_3_ (50 mL). The organic phase was washed with 2% HCl aq. (50 mL) for twice and brine, dried over Na_2_SO_4_. After filtration, volatiles were removed under reduced pressure. The resulting crude residue was dissolved THF (10 mL), H_2_O (1.0 mL), and MeOH (1.0mL) at room temperature. To the resulting solution, the mixture was added NaSH (70%, 360 mg, 4.50 mmol) at room temperature and stirred at 60 °C. After stirring for 22 h, the reaction mixture was cooled to room temperature and added AcOH (1.0 mL). After stirring an additional 1 h, the reaction mixture was concentrated under reduced pressure. The resulting residue was purified by silica gel column chromatography (CHCl_3_ : MeOH = 95 : 5 to 80 : 20) to give **2** as a yellow solid (198 mg, 0.695 mmol, 70%, 2 steps).

Yellow solid; M.p. 235-238 °C; ^1^H MNR (400 MHz, DMSO-*d*_6_): δ 14.30-14.05 (br, 1H), 14.00-13.70 (br, 1H), 8.08 (s, 1H), 5.08-5.00 (br, 1H), 4.94 (d, *J* = 6.8 Hz, 1H), 5.00-4.80 (br, 2H), 4.47 (q, *J* = 5.6 Hz, 1H), 4.07 (q, *J* = 4.2 Hz, 1H), 3.88 (q, *J* = 4.0 Hz, 1H), 3.65-3.54 (br, 1H), 3.54-3.42 ppm (m, 1H); ^13^C NMR (100 MHz, DMSO-*d*_6_): δ 171.3, 144.9, 142.2, 136.3, 131.4, 85.4, 77.5, 74.4, 71.9, 62.4 ppm; IR (KBr): *ν* 3364, 3290, 2944, 1608, 1575, 1559, 1526, 1386, 1219, 1117, 1083, 888, 709, 573 cm^–1^; HRMS (ESI) Anal. calcd. for C_10_H_13_N_4_O_4_S *m*/*z* 285.0652 [M+H]^+^, found 285.0656; [α]_D_^24^ –64.0 (*c* 0.135, DMSO).

(2*R*,3*S*,4*R*,5*S*)-2-(Hydroxymethyl)-5-(7-(methylthio)-1*H*-pyrazolo[4,3-*d*]pyrimidin-3-yl)tetrahydrofuran-3,4-diol (**3**)

To a stirred suspension of **2** (4.26 g, 15.0 mmol) in H_2_O (30 mL) were added NaOH (660 mg, 16.5 mmol) and methyl iodide (1.40 mL, 22.5 mmol) at 0 °C, and the mixture was stirred at the same temperature. After stirring for 1 h, the reaction temperature was raised to room temperature and stirred for an additional 2 h. The reaction mixture was quenched with 10% HCl aq. (5 mL), pH was adjusted to 6, and volatiles were removed under reduced pressure. The resulting residue was suspended in CHCl_3_ : MeOH = 6 : 1, the solution was filtered through a pad of silica gel. The filtrate was concentrated under reduced pressure, and the resulting residue was washed with CH_3_CN (100 mL) and acetone (80 mL), respectively. The resulting residue was purified by silica gel column chromatography (CHCl_3_ : MeOH = 100 : 0 to 90 : 10) to give **3** as a colorless solid (3.49 g, 11.7 mmol, 78%).

Colorless solid; M.p. 183-185 °C; ^1^H MNR (400 MHz, DMSO-*d*_6_) δ 14.08 (s, 1H), 8.78 (s, 1H), 5.14 (dd, *J* = 8.0, 4.4 Hz), 5.03 (d, *J* = 6.4 Hz, 1H), 4.95 (d, *J* = 4.8 Hz, 1H), 5.55 (dd, *J* = 12.0, 6.8 Hz, 1H), 4.12 (q, *J* = 4.8 Hz, 1H), 3.92 (q, *J* = 4.0 Hz, 1H), 3.65 (dt, *J* = 12.0, 4.0 Hz, 1H), 3.51 (ddd, *J* = 12.0, 8.0, 4.0 Hz, 1H), 2.74 ppm (s, 3H); ^13^C NMR (100  MHz, DMSO-*d*_6_): δ 155.1, 149.9, 144.3, 138.0, 130.6, 85.7, 77.7, 74.6, 72.0, 62.4, 11.2 ppm; IR (KBr): *ν* 3276, 3125, 3046, 2924, 2868, 2832, 1592, 1541, 1498, 1455, 1373, 1119, 1077, 1056, 1033, 943, 880, 834, 588 cm^–1^; HRMS (ESI) Anal. calcd. for C_11_H_15_N_4_O_4_S *m*/*z* 299.0809 [M+H]^+^, found 299.0811; [α]_D_^24^ –71.5 (*c* 0.155, DMSO).

((3a*R*,4*R*,6*S*,6a*S*)-2,2-Dimethyl-6-(7-(methylthio)-1*H*-pyrazolo[4,3-*d*]pyrimidin-3-yl)tetrahydrofuro[3,4-d][1,3]dioxol-4-yl)methanol (**4**)

To a stirred solution of **3** (298 mg, 1.00 mmol) in dimethylformamide (2.5 mL) were added 2,2-dimethoxy propane (614 µL, 5.00 mmol) and *p*-toluenesulfonic acid monohydrate (228 mg, 1.20 mmol) and the mixture was stirred at room temperature for 1 h. The reaction was quenched with sat. NaHCO_3_ aq. (10 mL), the resulting mixture was extracted with EtOAc (10 mL) three times. The organic phase was washed with 80% NaCl aq. and dried over Na_2_SO_4_. After filtration, volatiles were removed under reduced pressure. The resulting residue was purified by silica gel column chromatography (CHCl_3_ : EtOAc = 95 : 5 to 85 : 15) to give **4** as a white solid (315 mg, 0.930 mmol, 93%).

White solid; ^1^H MNR (400 MHz, CDCl_3_): δ 11.88-10.89 (br, 1H), 8.78 (s, 1H), 6.63-6.23 (br, 1H), 5.39 (d, *J* = 5.6 Hz, 1H), 5.08 (dd, *J* = 6.0, 1.6 Hz, 1H), 5.00 (t, *J* = 6.0 Hz, 1H), 4.54 (d, *J* = 1.6 Hz, 1H), 4.04 (dd, *J* = 12.8, 2.0 Hz, 1H), 3.89-3.75 (br, 1H), 2.76 (s, 3H), 1.67 (s, 3H), 1.38 ppm (s, 3H).

((3a*R*,4*R*,6*S*,6a*S*)-2,2-Dimethyl-6-(7-(methylthio)-1*H*-pyrazolo[4,3-*d*]pyrimidin-3-yl)tetrahydrofuro[3,4-*d*][1,3]dioxol-4-yl)methyl hydrogen phosphonate triethylamine salt (**5**) ^ref-SMe2^

To a stirred solution of **4** (135 mg, 0.400 mmol) in pyridine (2.0 mL) and 1,4-dioxane (2.0 mL) were 2-chloro-1,3,2-benzodioxaphosphorin-4-one (243 mg, 1.20 mmol) at 0 ^o^C, and the mixture was stirred at the same temperature for 1 h. The reaction was quenched with H_2_O (1.0 mL) and stirred for 10 min. The resulting mixture was concentrated under reduced pressure. The resulting residue was purified by silica gel column chromatography (CHCl_3_ : MeOH : Et_3_N = 95 : 5 : 1 to 90 : 10 : 1) to give **5** as an amorphous (165 mg, 0.233 mmol, 58%).

Amorphous; ^1^H MNR (400 MHz, CDCl_3_): δ 12.20-11.77 (br, 1H), 8.74 (s, 1H), 6.93 (d, *J* = 636 Hz, 1H), 5.57 (d, *J* = 4.4 Hz, 1H), 5.21-5.10 (br, 1H), 4.96 (dd, *J* = 6.4, 2.8 Hz, 1H), 4.37 (q, *J* = 3.2 Hz, 1H), 4.26 (td, *J* = 10.8, 3.2 Hz, 1H), 4.04 (td, *J* = 11.2, 4.0 Hz, 1H), 3.02 (q, *J* = 7.2 Hz, 6H), 2.68 (s, 3H), 1.63 (s, 3H), 1.36 (s, 3H), 1.22 ppm (t, *J* = 7.2 Hz, 9H); ^31^P NMR (162 MHz, CDCl_3_): δ 5.04 ppm (d, *J* = 637 Hz, 1P).

((3a*R*,4*R*,6*S*,6a*S*)-2,2-Dimethyl-6-(7-(methylthio)-1*H*-pyrazolo[4,3-*d*]pyrimidin-3-yl)tetrahydrofuro[3,4-*d*][1,3]dioxol-4-yl)methyl tetrahydrogen triphosphate triethylamine salt (**6**) ^ref-SMe3^

To a stirred solution of **5** (165 mg, 0.233 mmol) in dimethylformamide (2.3 mL) were added pyridine (470 µL, 5.84 mmol) and trimethylsilyl chloride (148 µL, 1.17 mmol) at 0 ^o^C, and the mixture was stirred at room temperature. After stirring for 30 min, 0.2 M I_2_ in DMF (1.4 mL, 0.280 mmol) was added. After stirring additional 5 min, tris(tetrabutylammonium) hydrogen pyrophosphate (316 mg, 0.350 mmol) was added and stirred additional 2 h. The reaction mixture was quenched with 10% Na2SO3 aq. (4.0 mL) and 1.0 M triethylammonium bicarbonate aq. (TEAB aq, 4.0 mL), and stirred an additional 10 min. The resulting mixture was concentrated under reduced pressure, and the resulting residue was purified by DEAE sephadex ^®^ (0.1 M TEAB aq. to 0.5 M TEAB aq.) to give **6** as an amorphous (53.9 mg, 45.5 µmol, 19%).

Amorphous; ^1^H MNR (400 MHz, D_2_O): δ 8.63 (s, 1H), 5.51-5.44 (m, 2H), 5.14 (dd, *J* = 6.0, 3.2 Hz, 1H), 4.55-4.46 (br, 1H), 4.08 (t, *J* = 5.6 Hz, 2H), 3.40-2.94 (m, 36H), 2.72 (s, 3H), 1.69 (s, 3H), 1.49 (s, 3H), 1.27 ppm (t, *J* = 7.2 Hz, 54H); ^31^P NMR (162 MHz, D_2_O): δ –10.12 (dd, *J* = 131, 19.3 Hz, 1P), –11.14 (dd, *J* = 119, 19.8 Hz, 1P), –22.74-–23.66 ppm (m, 1P).

Sodium ((2*R*,3*S*,4*R*,5*S*)-3,4-dihydroxy-5-(7-(methylthio)-1*H*-pyrazolo[4,3-*d*]pyrimidin-3-yl)tetrahydrofuran-2-yl)methyl triphosphate (**7**)

To a stirred solution of **6** (53.9 mg, 45.5 µmol) in H_2_O (341 µL) were added trifluoroacetic acid (114 µL) at 0 ^o^C, and the mixture was stirred at room temperature. After stirring for 30 min, the reaction mixture was cooled to 0 ^o^C, and the reaction was quenched with 1.0 M triethylammonium bicarbonate aq. (1.0 mL). The resulting mixture was concentrated under reduced pressure, and the resulting residue was purified by DEAE sephadex ^®^ (H_2_O to 0.5 M TEAB aq.). The fraction containing the triphosphate were combined, concentrated under reduced pressure. Further purification was achieved by preparative reverse-phase HPLC (Develosil RPAQUEOUS C30-UG 4.6x250mm, mobile phase: phase A: 10 mM of TEAB aq., pH = 7.5 adjusted with acetic acid; phase B: 10 mM of TEAB in CH_3_CN, pH = 7.5 adjusted with acetic acid) with a linear gradient of 0% to 20% of phase B over 25 min; retention time = 10.8~12.2 min, detected by absorbance at 254 nm, to give **7**-free acid as an amorphous. The triphosphate was dissolved in water, and the solution was passed through on Dowex 50Wx2 Na form, to give **7** as amorphous (12.9 mg, 20.6 µmol, 45%).

Amorphous; ^1^H MNR (400 MHz, D_2_O): δ 8.65 (s, 1H), 5.39 (d, *J* = 7.2 Hz, 1H), 4.79-4.73 (m, 1H), 4.50 (dd, *J* = 5.2, 4.4 Hz, 1H), 4.35 (q, *J* = 4.0 Hz, 1H), 4.28-4.17 (m, 2H), 2.73 ppm (s, 3H); ^31^P NMR (162 MHz, D_2_O): δ –9.60 (d, *J* = 18.6 Hz, 1P), –10.96 (d, *J* = 19.1 Hz, 1P), –22.70 ppm (t, *J* = 18.5 Hz, 1P).

Ref-SMe1 Miles, R.W.; Samano, V.; Robins, M.J. Nucleic Acid Related Compounds. 86. Nucleophilic Functionalization of Adenine, Adenosine, Tubercidin, and Formycin Derivatives via Elaboration of the Heterocyclic Amino Group into a Readily Displaced 1,2,4-Triazol-4-yl Substituent. *J. Am. Chem. Soc* **1995**, *117*, 5951–5957.

Ref-SMe2 Marugg, J.E.; Tromp, M.; Kuylyeheskiely, E.; van der Marel, G.A.; van Boom, J.H. A convenient and general approach to the synthesis of properly protected d-nucleoside-3′-hydrogenphosphonates *via* phosphite intermediates. *Tetrahedron Lett.* **1986**, *27*, 2661-2664.

Ref-SMe3 Sun, Q; Edathil, J.P.; Wu, R.; Smidansky E.D.; Cameron, C.E.; Peterson, B.R. One-Pot Synthesis of Nucleoside 5′-Triphosphates from Nucleoside 5′-*H*-Phosphonates. *Org. Lett.* **2008**, *10*, 1703–1706.
